# Clinical significance of toxin EIA positivity in patients with suspected *Clostridioides difficile* infection: systematic review and meta-analysis

**DOI:** 10.1128/jcm.00977-24

**Published:** 2024-12-12

**Authors:** Giannoula S. Tansarli, Matthew E. Falagas, Ferric C. Fang

**Affiliations:** 1 Department of Laboratory Medicine and Pathology, University of Washington School of Medicine 7284 , Seattle, Washington, USA; 2 Department of Medicine, Alfa Institute of Biomedical Science 69121 , Athens, Greece; 3 Department of Medicine, School of Medicine, European University Cyprus 486437 , Nicosia, Cyprus; 4 Clinical Microbiology Laboratory, Harborview Medical Center 21618 , Seattle, Washington, USA; 5 Department of Microbiology, University of Washington School of Medicine 312771 , Seattle, Washington, USA; Universität Münster, Münster, Germany

**Keywords:** *Clostridioides difficile*, CDI, toxin, diagnosis, EIA, enzyme immunoassay

## Abstract

The laboratory diagnosis of *Clostridioides difficile* infection (CDI) is controversial. Nucleic acid amplification tests (NAAT) and toxin enzyme immunoassays (EIA) are most widely used, often in combination. However, the interpretation of a positive NAAT and negative toxin immunoassay (NAAT+/EIA−) is uncertain. PubMed and EMBASE were searched for studies reporting clinical outcomes in NAAT+/EIA− versus NAAT+/EIA+ patients. Forty-six studies comprising 33,959 patients were included in this meta-analysis. All-cause mortality (RR 0.96, 95% CI 0.80–1.15), attributable mortality (RR 0.61, 95% CI 0.20–1.91), fulminant CDI (RR 0.83, 95% CI 0.57–1.20), radiographic evidence of CDI (RR 0.87, 95% CI 0.65–1.16), total CDI complications (RR 0.95, 95% CI 0.59–1.53), colectomies (RR 0.78, 95% CI 0.34–1.79), and ICU admission (RR 1.04, 95% CI 0.84–1.30) did not significantly differ between NAAT+/EIA− and NAAT+/EIA+ patients. However, rates of recurrent (RR 0.62, 95% CI 0.50–0.77) or severe (RR 0.74, 95% CI 0.63–0.88) CDI were significantly lower in NAAT+/EIA− patients than in NAAT+/EIA+ patients. The pooled prevalence of NAAT+/EIA− patients who were treated with antibiotics for CDI was 73.4% (pooled proportion 0.72, 95% CI 0.52–0.88). NAAT+/EIA− patients have lower rates of recurrence and are at reduced risk for severe CDI compared with NAAT+/EIA+ patients but have a risk of CDI-related complications and mortality comparable to that of NAAT+/EIA+ patients. Toxin results cannot rule in or rule out CDI, and the decision whether to treat symptomatic NAAT+/EIA− patients for CDI should be based on clinical presentation and not on the toxin result.

**IMPORTANCE:**

*Clostridioides difficile* infection (CDI) is a common cause of healthcare-associated infections and the leading cause of antibiotic-associated diarrhea. However, the laboratory diagnosis of CDI, primarily done by nucleic acid amplification test (NAAT) and enzyme immunoassay (EIA), is controversial, especially in patients who test positive by NAAT but negative by EIA. In this systematic review, we compared the clinical outcomes of NAAT+/EIA− versus NAAT+/EIA+ patients and found that the two groups have similar risk of mortality and CDI-related complications. However, NAAT+/EIA− patients had significantly lower rates of recurrence and severe CDI than NAAT+/EIA+ patients, and most NAAT+/EIA− patients received CDI therapy. Toxin testing can help to predict the likelihood of CDI recurrence or severe infection, but the toxin result should not be a determining factor in the administration of CDI therapy. The decision on whether to treat NAAT+/EIA− patients should be based on clinical assessment.

## INTRODUCTION

*Clostridioides difficile* infection (CDI) is the leading cause of antibiotic-associated infectious diarrhea worldwide (1, 2), resulting in significant morbidity and mortality (3–5). Although advances in CDI treatment have included new antimicrobial agents (6) and fecal microbiota transplantation (7), the laboratory diagnosis of CDI has remained a challenge (8). Toxigenic culture (culture combined with a tissue culture cytotoxin assay) is the traditional gold standard method against which new assays for *C. difficile* are compared but is of low clinical utility due to a long turnaround time. Enzyme immunoassays (EIAs) allowing the rapid detection of toxins A and B have been widely used despite suboptimal sensitivity. Since 2010, when the first nucleic acid amplification test (NAAT) for toxigenic *C. difficile* was approved by the FDA, many healthcare facilities replaced EIAs with rapid and highly sensitive NAATs (9).

However, *C. difficile* can be associated with a broad range of clinical presentations*,* ranging from asymptomatic colonization to severe colitis. Diagnosis therefore requires the laboratory detection of toxin or a toxigenic organism combined with the characteristic clinical features of CDI. NAATs are considered more sensitive but less specific, whereas EIAs are more specific (10) but less sensitive (11), potentially resulting in overdiagnosis or underdiagnosis, respectively (12–16). Many healthcare institutions employ a combination of tests in multistep algorithms that include both NAAT and EIA.

CDI is a laboratory-identified event in the USA that must be reported to the National Healthcare Safety Network (NHSN). The NHSN revised its CDI reporting guidelines in 2018, permitting institutions using multistep algorithms to report only the last positive CDI result (17). Following this change, some US healthcare institutions that formerly relied on NAAT-only to diagnose CDI switched to a two-step algorithm consisting of NAAT with reflexive EIA in the case of a positive NAAT result. In Europe and other parts of the world, two-step algorithms combining NAAT with toxin EIA have also been widely used in accordance with guidelines by the European Society of Clinical Microbiology and Infectious Diseases (ESCMID) and the Infectious Diseases Society of America (IDSA) (15, 18), primarily to reduce overtreatment of toxin-negative patients who may be merely colonized (18). However, the use of the two-step algorithm can lower reported institutional CDI rates while underestimating actual infection rates. Moreover, clinicians may be uncertain about how to manage symptomatic patients with discordant NAAT+/EIA− test results.

In the present study, we performed a systematic review and meta-analysis of the published literature to evaluate the clinical outcomes of patients who are NAAT-positive but negative by toxin EIA (NAAT+/EIA−) compared to those who test positive by both NAAT and EIA (NAAT+/EIA+) to better inform clinical test interpretation.

## MATERIALS AND METHODS

### Literature search

PubMed and Embase databases were systematically searched for eligible studies by two independent reviewers (GST and FCF) until the end of February 2024; no backward time limit was set. Any disagreement between reviewers regarding the relevance of a study was resolved by consensus. The following search terms were applied to both databases without a year limit: (“*Clostridium difficile*” OR “*Clostridioides difficile*”) AND (ELISA OR toxin OR EIA OR “enzyme immunoassay”) AND (NAAT OR “nucleic acid amplification test” OR PCR) AND (outcome OR treatment OR mortality OR recurrence OR relapse OR surgical OR surgery OR complication). The bibliographies of the included studies were also hand-searched for potentially eligible studies. Only articles published in English were evaluated. Unpublished data (i.e., from abstracts presented at conferences) and studies on pediatric patients were excluded from this review. This systematic review and meta-analysis was performed in accordance with the *Cochrane Handbook for Systematic Reviews of Interventions* and PRISMA guidelines (19, 20).

### Study selection

Any study comparing outcomes in CDI patients who were NAAT+/EIA− vs NAAT+/EIA+ was considered eligible for inclusion in the meta-analysis. Other eligible studies were those comparing outcomes between NAAT + or toxigenic culture-positive/EIA− and EIA+ CDI patients as well as those comparing outcomes in NAAT+ vs EIA+. Each of these groups of studies was analyzed separately. Additional analyses were performed among CDI patients who were NAAT+/EIA− to compare outcomes between those who received treatment for CDI vs those who were not treated. Lastly, studies that followed the two-step algorithm were pooled, with receipt of antibiotics for CDI compared between NAAT+/EIA− and NAAT+/EIA+ groups. The quality of the included studies was assessed using the Newcastle-Ottawa Scale (NOS) for cohort studies (21).

### Data extraction and definitions

The following variables were extracted from each study: author name, year of publication, study design, NOS score, number of included patients, NAAT and EIA assays used for the detection of *C. difficile* and toxins, clinical outcomes (all-cause mortality, CDI recurrence, fulminant CDI, severe CDI, total complications, colectomies, radiographic evidence of CDI, ICU admission due to CDI or shortly after CDI diagnosis, attributable mortality) in each patient group, and proportion of NAAT+/EIA− patients receiving antibiotic treatment for CDI.

Recurrence was defined as either readmission or retesting for CDI within 3 months. For fulminant CDI (also referred to as “complicated” in some studies), only studies that followed the IDSA/SHEA definition (i.e., presence of hypotension or shock, ileus, or megacolon) (22) or defined fulminant CDI as needing surgical intervention were pooled together. Similarly, for severe CDI, only studies following the IDSA/SHEA definition (i.e., WBC count of >15,000 cells/mL or serum creatinine level ≥1.5 mg/dL) (22) were pooled together. Studies that presented separate numbers for toxic megacolon, ileus, or surgery other than colectomy were pooled in an analysis called “total complications,” while studies that provided numbers of colectomies following CDI in NAAT+/EIA− vs NAAT+/EIA+ groups were pooled in a separate analysis.

To ensure the quality of the meta-analysis, only published data from each study were analyzed.

### Meta-analysis

The meta-analysis was performed with Review Manager for Windows, version 5.4.1. Pooled risk ratios (RR) and 95% confidence intervals (CI) were calculated for all outcomes. Statistical heterogeneity among studies was assessed by using a *χ*^2^ test (*P* < 0.10 was defined to indicate significant heterogeneity) and *I*^2^. Because of differences in the populations described by the included studies, a random effects model with the DerSimonian and Laird approach was used in all analyses (23).

The following patient groups were compared in terms of the aforementioned outcomes depending on available data in each study: NAAT+/EIA− vs NAAT+/EIA+, NAAT+ or toxigenic culture-positive/EIA− vs EIA+, NAAT+ vs EIA+, and treated vs untreated NAAT+/EIA− patients. The R packages meta and metafor were used for this type of pairwise meta-analysis. In addition, studies that provided the percentage of NAAT+/EIA− patients receiving antibiotic treatment for CDI were pooled together in a proportional meta-analysis. For the proportional meta-analysis, the metaprop function from the R meta package was used (24).

## RESULTS

**Fig 1:**
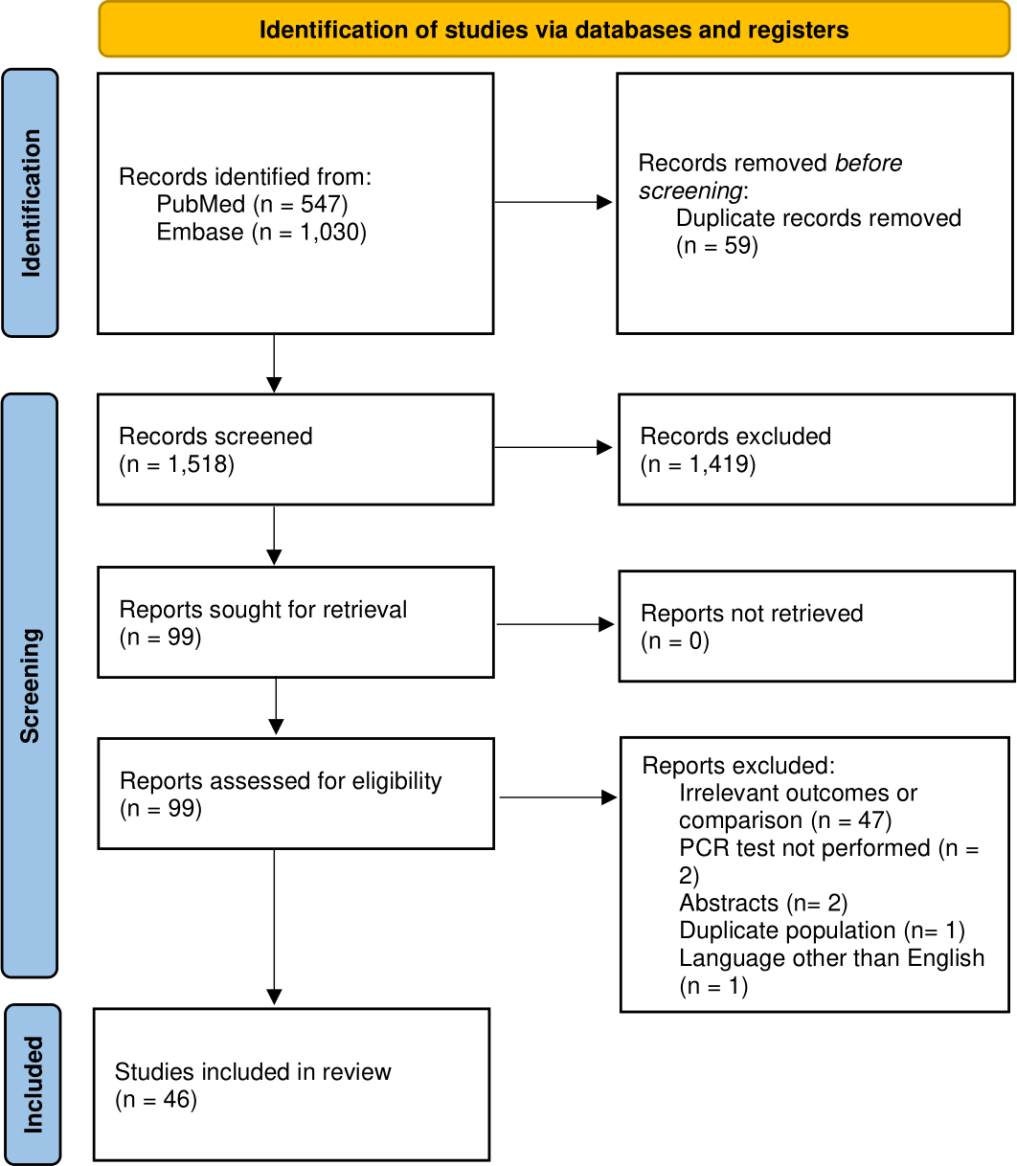
Flow diagram of the systematic search and study selection process.

A systematic search of both databases generated 1,577 results (PubMed 547, EMBASE 1,030), of which 46 studies (33,959 cases) were included in the analysis. One study reported pediatric patients and was excluded (25), and another (26) was excluded because the study population overlapped with another study from the same research group (27). The detailed study selection process is depicted in the PRISMA flow diagram (Fig. 1), and characteristics of the included studies are presented in Table 1.

**TABLE 1:** Characteristics of the studies included in the meta-analysis

Author, year	Country	Study design	NOS score	Number of cases included; study aim	Compared groups	Assays used	Studied outcomes
Guh, 2024 (28)	USA	Retrospective	9*	1,801; evaluation of the two-step algorithm	NAAT+/EIA− vs NAAT+/EIA+ and treated vs untreated NAAT+/EIA−	NAATs: Cepheid Xpert *C*. *difficile* (LoD: 460 cfu/swab); Meridian Bioscience illumigene *C*. *difficile* Test Kit (LoD: 4–64 CFU/reaction); Simplexa *C*. *difficile* Direct (LoD: 0.95 cfu/mL). EIAs: Wampole Tox A/B Quik Chek (Techlab) (LoD: 0.63 ng/mL for toxin A, 0.16 ng/mL for toxin B); Meridian Bioscience Premier toxins A&B (LoD: toxin A ≥ 1.4 ng/mL and toxin B ≥ 2.4 ng/mL stool); Techlab Tox A/B Quik Chek (0.63 ng/mL toxin A and 1.25 ng/mL toxin B); Techlab *C*. *difficile* Tox A/B II (LoD: toxin A ≥ 0.8 ng/mL and toxin B ≥ 2.5 ng/mL); Meridian Bioscience Immunocard Toxins A&B (LoD: 3 ng of toxin A and 3 ng of toxin B per mL).	Recurrence, fulminant CDI, ICU admission
Bettger, 2023 (29)	USA	Retrospective	7*	216; evaluation of the two-step algorithm	NAAT+/EIA− vs NAAT+/EIA+ and treated vs untreated NAAT+/EIA−	NAAT: Xpert *C. difficile* (Cepheid) EIA: Wampole *C*. *difficile* Testing, Tox A/B II EIA 96 T	Mortality, recurrence
Dbeibo, 2023 (30)	USA	Retrospective	8*	610; evaluation of the two-step algorithm	NAAT+/EIA− vs NAAT+/EIA+ and treated vs untreated NAAT+/EIA−	NR	Mortality, ICU admission
Hecker, 2023 (31)	USA	Retrospective	7*	85; evaluation of the two-step algorithm	NAAT+/EIA− vs NAAT+/EIA+	NAAT: NR EIA: TOX A/B QUIK CHEK enzyme immunoassay for toxins A and B (Alere)	Recurrence, attributable mortality
Pender, 2023 (32)	USA	Retrospective	8*	444; evaluation of positive GI multiplex PCR results	NAAT+/EIA− vs NAAT+/EIA+	NAAT: Biofire FilmArray assay (LoD: 4 × 10^5^ cells/mL) EIA: toxin A/B by enzyme immunoassay (Quik Chek Complete; TechLab)	ICU admission
Turner, 2023 (33)	USA	Prospective (longitudinal cohort)	No individual patient data	13,486; evaluation of the two-step algorithm	No comparison in outcomes	NR	Proportion of treated NAAT+/EIA− patients
Evans, 2022 (34)	USA	Retrospective	7*	2,374; determine how often NAAT+/EIA− patients are treated	No comparison in outcomes	NR	Proportion of treated NAAT+/EIA− patients
Johnson, 2022 (35)	USA	Retrospective	8*	71; treatment of NAAT+/EIA− IBD patients	NAAT+/EIA− vs NAAT+/EIA+ and treated vs untreated NAAT+/EIA−	NR	Mortality, recurrence, fulminant CDI
Kim, 2022 (36)	South Korea	Retrospective	8*	120; adherence to CDI treatment guidelines	NAAT+/EIA− vs NAAT+/EIA+ and treated vs untreated NAAT+/EIA−	NAAT: BD MAX Cdiff assay (BD Diagnostics) (LoD: 125 to 265 CFU per loop) EIA: C. DIFF QUIK CHEK COMPLETE (TechLab)	Mortality, recurrence, ICU admission, radiographic evidence of CDI
Sopena, 2022 (37)	Spain	Retrospective	9*	443; evaluation of the two-step algorithm	NAAT+/EIA− vs NAAT+/EIA+	NAAT: GenomEra CD (Abacus Diagnostica) EIA: C. DIFF QUIK CHEK COMPLETE test (Abbott)	Mortality, recurrence, fulminant CDI
Guevara, 2021 (38)	USA	Prospective	6*	227; evaluation of the two-step algorithm in cancer patients	NAAT+/EIA− vs NAAT+/EIA+	NAAT: Biofire FilmArray assay EIA: Immunocard *C. difficile* A&B assay (Meridian Bioscience)	Mortality, recurrence, severe CDI
Gupta, 2021 (39)	USA	Retrospective	8*	64; assess treatment response in IBD patients who were NAAT+/EIA−	No comparison in outcomes	NAAT & EIA: TechLab assays (Blacksburg, VA)	Proportion of treated NAAT+/EIA− patients
Herman, 2021 (40)	Canada	Retrospective	7*	364; evaluate treatment of NAAT+/EIA− patients after change in result reporting	Treated vs untreated NAAT+/EIA−	NR	Mortality
Turner, 2021 (41)	Australia	Retrospective	4*	200; evaluation of the two-step algorithm	NAAT+ vs EIA+	NR	Recurrence
Honda, 2020 (42)	Japan	Prospective	8*	131; risk factors for CDI	NAAT+ or TC+/EIA− vs EIA+	EIAs: C. Diff Quik Chek Complete, ImmunoCard Toxins A&B (Meridian Bioscience); GE test Immuno-Chromato CD TOX A/B (Nissui Pharmaceuticals)	Mortality, recurrence
Lenggenhager, 2020 (43)	Switzer- land	Retrospective	8*	208; identify the proportion and characteristics of NAAT/EIA− who receive treatment for CDI	No comparison in outcomes	NAAT: BD MAX (Becton-Dickinson) EIA: XPect *C. difficile* Toxin A/B EIA (Remel) (LoD: toxin A at >6.25 ng/mL and toxin B at >40.0 ng/mL)	Proportion of treated NAAT+/EIA− patients
Miller, 2020 (44)	USA	Retrospective	8*	352; evaluation of the two-step algorithm	NAAT+/EIA− vs NAAT+/EIA+ and treated vs untreated NAAT+/EIA−	NAAT: Xpert *C. difficile* test (Cepheid) EIA: Tox A/B Quik Chek (TechLab)	Mortality, recurrence, fulminant CDI, severe CDI, ICU admission, attributable mortality
Olmedo, 2020 (45)	Spain	Prospective	7*	146; evaluate different *C*. *difficile* laboratory tests	NAAT+/EIA− vs NAAT+/EIA+	NAAT: Xpert *C*. *difficile* Assay, GeneXpert (Cepheid) EIA: CDiff Quik-Chek Complete Assay (TechLab)	Mortality, recurrence, fulminant CDI, attributable mortality
Shah, 2020 (46)	USA	Prospective	7*	80; compare different *C*. *difficile* laboratory tests	NAAT+/EIA− vs NAAT+/EIA+	NAAT: Xpert *C. difficile*/Epi assay (Cepheid) EIAs: C. diff Quik Chek Complete (TechLab); Premier toxins A and B (Meridian Bioscience); Singulex Clarity C. diff toxins A/B assay	Mortality, recurrence, severe CDI, radiographic evidence of CDI
Vogelzang, 2020 (47)	Nether- lands	Retrospective	7*	31; assess frequency of treatment in NAAT+/EIA− patients	No comparison in outcomes	NAAT: In-house real-time PCR EIA: ImmunoCard toxins A and B (Meridian Bioscience)	Proportion of treated NAAT+/EIA− patients
Wadskier, 2020 (48)	USA	Prospective	9*	119; evaluate the clinical significance of a positive *C*. *difficile* result by GI multiplex PCR	NAAT+ vs EIA+	NAAT: Biofire FilmArray assay EIA: C. Diff Quik Chek Complete, Abbott	Mortality, recurrence
Guh, 2019 (49)	USA	Retrospective	9*	4,878; compare outcomes of patients diagnosed by NAAT vs EIA	NAAT+/EIA− vs NAAT+/EIA+	NAAT: Not specified EIA: C. Diff Quik Chek Complete (Techlab)	Mortality, recurrence, fulminant CDI, ICU admission
Hitchcock, 2019 (50)	USA	Retrospective	7*	482; evaluation of reporting of PCR cycle threshold for *C*. *difficile*	NAAT+/EIA− vs NAAT+/EIA+ and treated vs untreated NAAT+/EIA−	NAAT: Xpert C. diff/Epi *tcdB* PCR assay (Cepheid) EIA: Not specified	Mortality, fulminant CDI, attributable mortality
Pollock, 2019 (51)	USA	Prospective	7*	201; compare toxin concentrations in symptomatic vs asymptomatic patients	NAAT+/EIA− vs NAAT+/EIA+	NAAT: Xpert *C. difficile*/Epi (Cepheid) Toxin immunoassay: Simoa (LoD: toxin A 0.6 pg/mL and toxin B 2.9 pg/mL)	Mortality, ICU admission
Sandlund, 2019 (52)	USA	Prospective	7*	64; evaluate different *C*. *difficile* laboratory tests	NAAT+/EIA− vs NAAT+/EIA+	NAATs: BD MAX Cdiff assay, Xpert *C. difficile* assay (Cepheid) EIA: Singulex Clarity C. diff toxins A/B assay	Recurrence, ICU admission
Ziegler, 2018 (53)	USA	Retrospective	6*	182; compare outcomes of patients with hematologic malignancy diagnosed by NAAT vs EIA	NAAT+ vs EIA+	NAAT: BD MAX Cdiff Assay, Becton Dickinson EIA: C Diff Quik Check Complete, Alere	Mortality, recurrence, fulminant CDI, ICU admission
Avni, 2018 (54)	Israel	Retrospective	8*	322; compare outcomes of patients diagnosed by NAAT vs EIA	NAAT+ vs EIA+	NAAT: Xpert *C*. *difficile* PCR assay EIA: C.DIFF QUIK CHEK COMPLETE assay	Mortality, recurrence, fulminant CDI, ICU admission
Kim, 2018 (55)	South Korea	Retrospective	7*	282; evaluate the clinical significance of toxin positivity and toxin gene load	NAAT+/EIA− vs NAAT+/EIA+	NAAT: Xpert *C. difficile* system (Cepheid) EIA: VIDAS *C. difficile* toxin A&B (LoD: Toxin A > 7.73 ng/mL, toxin B > 4.55 ng/mL)	Mortality, fulminant CDI
Origuen, 2018 (56)	Spain	Retrospective	8*	231; compare outcomes of patients diagnosed by NAAT vs EIA	NAAT+/EIA− vs NAAT+/EIA+ and treated vs untreated NAAT+/EIA−	NAAT: GeneXpert *C*. *difficile* PCR assay (Cepheid) EIA: TechLab C. diff Quik Chek Complete	Mortality, recurrence, fulminant CDI, severe CDI, attributable mortality
Theiss, 2018 (57)	USA	Retrospective	8*	201; evaluate the addition of EIA testing in the testing algorithm for *C*. *difficile*	NAAT+/EIA− vs NAAT+/EIA+	NAAT: BD Max Cdiff assay EIA: Meridian ImmunoCard Toxins A and B assay	Mortality, fulminant CDI
Zou, 2018 (58)	Canada	Retrospective	7*	110; compare outcomes of patients diagnosed by NAAT vs EIA	Treated vs untreated NAAT+/EIA−	NAAT: In-house real-time PCR EIA: C.DIFF QUIK CHEK COMPLETE, TECHLAB	Mortality
Kumar, 2017 (59)	UK	Retrospective	7*	299; compare outcomes of patients diagnosed by NAAT vs EIA	NAAT+ or TC+/EIA− vs EIA+	NAAT: GeneXpert *C*. *difficile* (Cepheid) EIA: C.DIFF QUIK CHEK COMPLETE, TECHLAB	Mortality
Ramos-Martinez, 2016 (60)	Spain	Retrospective	8*	107; compare outcomes of patients diagnosed by NAAT vs EIA	NAAT+/EIA− vs NAAT+/EIA+	NAAT: GenomEra CDX *C. difficile* assay (Abacus Diagnostica) (LoD: not found) EIA: C. DiffQuikChek Complete Techlab	Mortality, recurrence, ICU admission
Reigadas, 2016 (61)	Spain	Prospective	7*	169; evaluate the clinical significance of direct cytotoxicity and toxigenic culture	NAAT+ or TC+/EIA− vs EIA+	NAAT: Xpert *C*. *difficile* assay, GeneXpert (Cepheid) EIA: C Diff Quik-Chek Complete assay (TechLab)	Mortality, recurrence, radiographic evidence of CDI, attributable mortality
Akbari, 2015 (62)	USA	Retrospective	6*	2,434; evaluate CDI diagnosis trends and outcomes after the introduction of NAAT	NAAT+ vs EIA+	NAAT: illumigene *C*. *difficile* (Meridian Bioscience) EIA: VIDAS *C*. *difficile* (bioMérieux)	Mortality
Erb, 2015 (63)	Switzer- land	Retrospective	7*	480; evaluate the addition of toxigenic culture in EIA− patients	NAAT+ or TC+/EIA− vs EIA+	EIA: CDIFF TOX A/B II; TechLab/ Wampole	Mortality, recurrence, fulminant CDI, ICU admission
Patel, 2015 (64)	USA	Retrospective	7*	168; compare clinical characteristics between toxin-positive and toxin-negative patients	NAAT+ or TC+/EIA− vs EIA+	NAAT: BD MAX Cdiff (GeneOhm Sciences) (LoD: 4 CFU/reaction or 10 copies per reaction) EIA: *C. Difficile* Quick Chek CompleteR (CdQ), (Techlab)	Mortality, recurrence, ICU admission
Polage, 2015 (16)	USA	Prospective	7*	293; determine the need to treat NAAT+/EIA− patients	NAAT+/EIA− vs NAAT+/EIA+	NAATs: Xpert *C*. *difficile*/Epi (Cepheid), illumigene *C*. *difficile* (Meridian Biosciences) EIA: *C*. *difficile* Premier toxins A and B (Meridian Biosciences)	Mortality, fulminant CDI, ICU admission, attributable mortality
Beaulieu, 2014 (27)	Canada	Retrospective	7*	86; compare outcomes of patients diagnosed by NAAT vs toxin-based (3-step) algorithm	NAAT+/EIA− vs NAAT+/EIA+	NAAT: BD GeneOhm Cdiff EIA: ToxA/B Quik-Check, Techlab	Mortality, recurrence, fulminant CDI, ICU admission
Baker, 2013 (65)	UK	Prospective	8*	77; compare clinical characteristics of patients diagnosed by NAAT vs EIA	NAAT+/EIA− vs NAAT+/EIA+	NAAT: Cepheid Xpert *C*. *difficile* assay EIA: Premier toxins A&B (Meridian Bioscience)	Mortality
Humphries, 2013 (66)	USA	Prospective	7*	143; compare outcomes of patients diagnosed by NAAT vs EIA	NAAT+/EIA− vs NAAT+/EIA+	NAAT: Cepheid Xpert *C. difficile* assay EIA: Premier Toxin A_B (Meridian Bioscience)	Mortality, recurrence, severe CDI, radiographic evidence of CDI
Planche, 2013 (67)	UK	Prospective	9*	746; develop an optimum laboratory diagnostic algorithm for *C*. *difficile* based on clinical outcomes	NAAT+/EIA− vs NAAT+/EIA+	NAAT: GeneXpert (Cepheid) EIAs: Meridian Premier toxins A&B enzyme immunoassay; Techlab *C*. *difficile* Tox A/B II	Mortality
Wang, 2013 (68)	USA	Retrospective	7*	27; compare outcomes of IBD patients diagnosed by NAAT vs EIA	NAAT+ vs EIA+	NAAT: Becton, Dickinson and Company EIA: Meridian Bioscience	ICU admission
Kaltsas, 2012 (69)	USA	Retrospective	6*	128; compare clinical characteristics of patients diagnosed by NAAT vs EIA	NAAT+/EIA− vs NAAT+/EIA+	NAAT: Xpert *C. difficile*/epi PCR (Cepheid) EIA: C. DIFF CHEK-60 (Techlab)	Mortality, recurrence ICU admission, radiographic evidence of CDI
Lee, 2012 (70)	South Korea	Prospective	7*	145; evaluation of the two-step algorithm	NAAT+ or TC+/EIA− vs EIA+	NAAT: PCR for *tcdB* (Applied Biosystems) EIA: assay by bioMérieux	Mortality, recurrence, severe CDI
Guerrero, 2011 (71)	USA	Prospective	7*	132; compare clinical characteristics of patients diagnosed by NAAT vs EIA	NAAT+/EIA− vs NAAT+/EIA+	NAAT: Becton Dickinson EIA: Wampole *C*. *difficile* TOX A/B II	Mortality, recurrence, fulminant CDI, severe CDI, attributable mortality

Thirty studies were retrospective cohort (27–32, 34, 35, 37, 39–41, 43, 44, 47, 49, 50, 53–60, 62–64, 68, 69), and 16 were prospective cohort design (16, 33, 38, 42, 45, 46, 48, 51, 52, 61, 65–67, 70, 71). Twenty-seven studies compared outcomes between NAAT+/EIA− and NAAT+/EIA+ patients (16, 27–32, 35–38, 44, 45, 49–52, 55–57, 60, 65–69, 71), six studies compared NAAT+ or toxigenic culture-positive/EIA− and EIA+ patients (42, 59, 61, 63, 64, 70), six studies compared NAAT+ and EIA+ patients (41, 48, 53, 54, 62, 68). Five studies that reported only the proportion of NAAT+/EIA− who received treatment for CDI were pooled in a separate analysis (33, 34, 39, 43, 47). Three studies included only patients with inflammatory bowel disease (35, 39, 68), and one study included only patients with hematologic malignancies (53). The remaining studies included CDI patients with mixed clinical characteristics. Over half of the included studies (26 of 46) were conducted in the USA. Seven studies provided data on the prevalence of hypervirulent strains (i.e., ribotype 027) (16, 38, 45, 49–51, 71). In four of them, the prevalence of ribotype 027 was significantly higher in EIA+ than in EIA− patients (16, 38, 49, 50, 71), while in two studies, the prevalence was numerically higher in EIA+ patients without reaching statistical significance (45, 51). Only one study reported outcomes based on *C. difficile* strain and found that patients with ribotype 027 had a significantly higher incidence of treatment failure (38). Five studies had medium quality (4–6 stars) based on the calculated NOS score (38, 41, 53, 62, 69), and one study did not provide individual patient data; thus, a NOS score could not be calculated (33). The remaining studies were considered of high quality based on the NOS score (7–9 stars).

### NAAT+/EIA− vs NAAT+/EIA+

**Fig 2:**
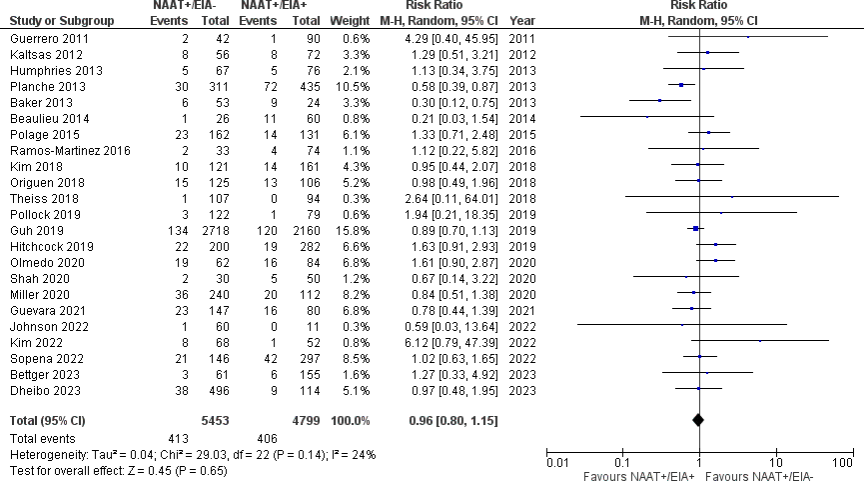
All-cause mortality in NAAT+/EIA– vs NAAT+/EIA+ patients.

Patients who were NAAT+/EIA– did not differ from those who were NAAT+/EIA+ with regard to risk of all-cause mortality (23 studies [16, 27, 29, 30, 35–38, 44–46, 49–51, 55–57, 60, 65–67, 69, 71]; Fig. 2; 10,252 patients; 7.6% vs 8.5%; RR 0.96, 95% CI 0.80–1.15, *I*^2^ 24%), fulminant CDI (12 studies [16, 27, 28, 35, 44, 45, 49, 50, 55–57, 71]; Fig. S1; 4,957 patients; 4.5% vs 5.9%; RR 0.83, 95% CI 0.57–1.20, *I*^2^ 26%), total complications (5 studies [28, 35, 44, 45, 56]; Fig. S2; 1,712 patients; 4.4% vs 3.9%; RR 0.95, 95% CI 0.59–1.53, *I*^2^ 0%), colectomies following CDI (4 studies [27, 28, 44, 49]; Fig. S3; 7,048 patients; 0.3% vs 0.4%; RR 0.78, 95% CI 0.34–1.79, *I*^2^ 0%), radiographic evidence of CDI (5 studies [36, 46, 49, 66, 69]; Fig. S4; 2,207 patients; 6.6% vs 8%; RR 0.87, 95% CI 0.65–1.16, *I*^2^ 0%), ICU admission (10 studies [27, 28, 30, 32, 36, 49, 51, 52, 60, 69]; Fig. S5; 8,436 patients; 5.7% vs 5.3%; RR 1.04, 95% CI 0.84–1.30, *I*^2^ 11%), or attributable mortality (7 studies [16, 31, 44, 45, 50, 56, 71]; Fig. S6; 1,654 patients; 1.8% vs 3.4%; RR 0.61, 95% CI 0.20–1.91, *I*^2^ 47%).

**Fig 3:**
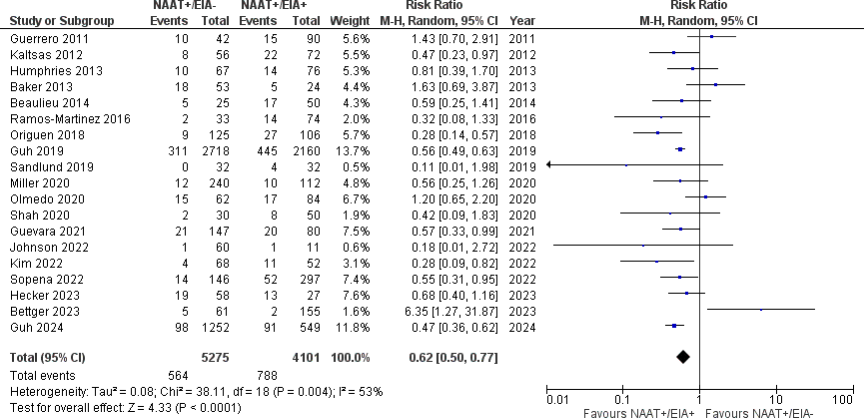
Recurrence in NAAT+/EIA– vs NAAT+/EIA+ patients.

**Fig 4:**
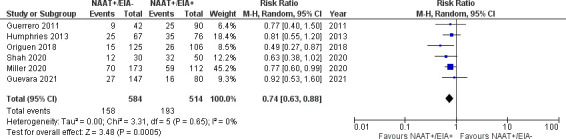
Severe CDI in NAAT+/EIA– vs NAAT+/EIA+ patients.

However, risk of recurrent (19 studies [27–29, 31, 35–38, 44–46, 49, 52, 56, 60, 65, 66, 69, 71]; Fig. 3; 9,376 patients; 10.7% vs 19.2%; RR 0.62, 95% CI 0.50–0.77, *I*^2^ 53%) or severe (6 studies [38, 44, 46, 56, 66, 71]; Fig. 4; 1,098 patients; 27.1% vs 37.5%; RR 0.74, 95% CI 0.63–0.88, *I*^2^ 0%) CDI was significantly lower in NAAT+/EIA– patients than NAAT+/EIA+ patients. Moreover, receipt of antibiotics for CDI was significantly less frequent in NAAT+/EIA– patients than in NAAT+/EIA+ patients (six studies (28–31, 50, 56); Fig. S7; 3,014 patients; 67.3% vs 93.6%; RR 0.70, 95% CI 0.61–0.79, *I*^2^ 88%).

### NAAT+ or toxigenic culture-positive/EIA– vs EIA+

No significant difference was observed for either all-cause mortality (six studies [42, 59, 61, 63, 64, 70]; Fig. S8; 1,382 patients; 7% vs 10%; RR 0.76, 95% CI 0.51–1.11, *I*^2^ 0%) or recurrence (five studies [42, 61, 63, 64, 70]; Fig. S9; 1,074 patients; 9% vs 11.7%; RR 0.87, 95% CI 0.44–1.72, *I*^2^ 55%].

### NAAT+ vs EIA+

No significant difference was observed for either all-cause mortality (four studies [48, 53, 54, 62]; 3,057 patients; 12.7% vs 10.6%; RR 0.80, 95% CI 0.49–1.31, *I*^2^ 68%) or recurrence (four studies [41, 48, 53, 54]; 823 patients; 13.2% vs 14.4%; RR 0.92, 95% CI 0.59–1.42, *I*^2^ 31%] between the compared groups.

### NAAT+/EIA– patients: treatment and outcomes

**Fig 5:**
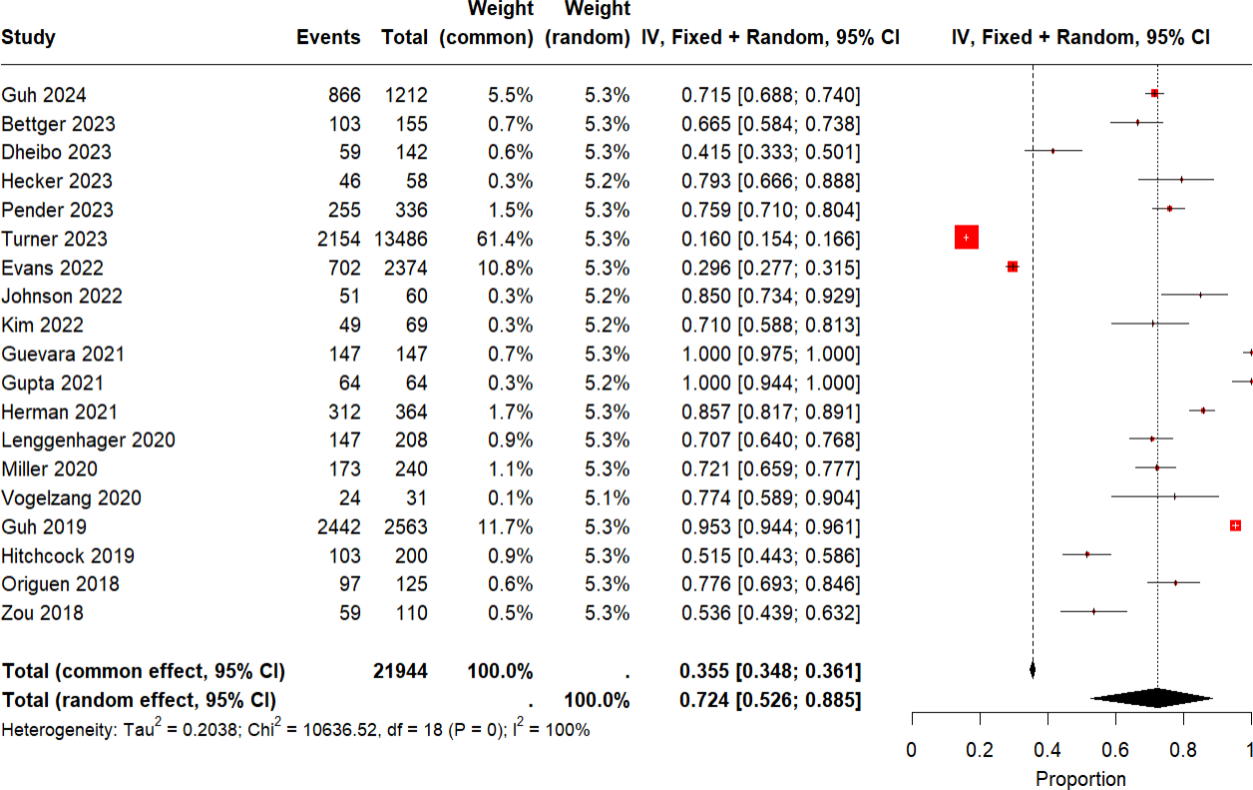
Pooled prevalence of CDI treatment in NAAT+/EIA– patients.

**Fig 6:**
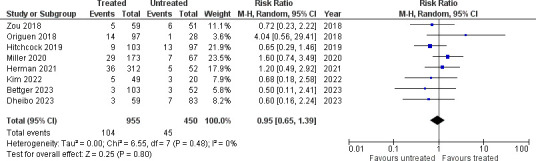
All-cause mortality in treated vs untreated NAAT+/EIA– patients.

We assessed the likelihood of antibiotic treatment for CDI in patients who were NAAT+/EIA– and the clinical outcomes in those who were treated compared to those who were not, and we pooled these data in a proportional meta-analysis. The pooled incidence of antibiotic treatment for CDI in NAAT+/EIA– patients was 73.4% (19 studies [28–36, 38–40, 43, 44, 47, 49, 50, 56, 58]; Fig. 5; 21,944 patients; pooled proportion 0.72, 95% CI 0.52–0.88, *I*^2^ 100%). Mortality (eight studies [29, 30, 36, 40, 44, 50, 56, 58]; Fig. 6; 1,405 patients; 10.9% vs 10%; RR 0.98, 95% CI 0.65–1.39, *I*^2^ 0%) was not significantly different between treated vs untreated patients.

## DISCUSSION

This systematic review and meta-analysis was undertaken to assess the clinical significance of toxin detection by EIA in patients undergoing testing for CDI. *C. difficile* carriage may be observed in 5%–15% of newly admitted patients and in as many as 51% of residents of long-term care facilities (72). It has been suggested that NAAT+/EIA− patients are asymptomatically colonized with *C. difficile* and do not require antibiotic treatment for CDI (73). However, this is clearly not the case, as more recent studies have shown that stool toxin assays cannot distinguish asymptomatic *C. difficile* colonization from symptomatic infection (51). Although there is a consensus that asymptomatic patients should not be tested for CDI, the role of toxin EIA in symptomatic patients, most of whom have diarrhea, is less clear. As many as 70% of patients undergoing two-step testing with NAAT and EIA have discordant results (28) with NAAT+ and EIA−. The goal of this study was to establish the clinical significance of these results based on a comprehensive review of the literature.

Our findings demonstrate that NAAT+/EIA+ patients have significantly higher rates of recurrence (1.7-fold) than NAAT+/EIA− patients. A higher risk of recurrence among toxin-positive patients is a highly consistent finding, suggesting that higher organism load (which correlates with toxin levels) is a risk factor for recurrence. Moreover, severe CDI is more frequent in EIA+ patients, suggesting that organism load is a risk factor for disease severity. This is supported by some studies correlating very low cycle threshold (Ct) values (which correlate with high toxin levels) with poor clinical outcomes (74–76). Although severe CDI, defined as WBC count of >15,000 cells/mL or serum creatinine level ≥1.5 mg/dL, was more frequent in EIA+ patients, more than a quarter of EIA− patients met criteria for severe CDI, indicating that a negative toxin result does not always translate to milder disease. The fact that EIA+ patients did not have higher rates of fulminant CDI, colectomies, ICU admission, or mortality compared with EIA− patients may be attributed to prompt initiation of appropriate antibiotic treatment. In institutions where two-step testing is performed, NAAT+/EIA+ patients are more likely to receive antibiotics for CDI than NAAT+/EIA− patients. However, it is unknown whether this is because NAAT+/EIA+ are more likely to warrant therapy or because clinicians regard EIA− positivity as an indication for therapy. Another consideration is that administration of antibiotics to NAAT+/EIA+ patients with collateral effects on the gut microbiota might be contributing to the higher recurrence rates noted in this group in comparison to NAAT+/EIA− patients.

In contrast, we did not find significant differences in all-cause or attributable mortality, fulminant infection, radiographic evidence of infection, or ICU admission between NAAT+/EIA− and NAAT+/EIA+ patients. From the 23 studies (>10,000 patients) included in the subgroup analysis for all-cause mortality, only two reported significantly higher mortality in NAAT+/EIA+ patients (65, 67). One of these studies included 746 patients and had the second highest weight in the all-cause mortality analysis (67), while the other included only 77 patients (65). The study with the highest weight in this analysis, which included 4,878 patients, showed no difference in mortality between toxin-positive and toxin-negative patients (49). The analysis of attributable mortality involved a much smaller sample (1,600 patients), and only 1 of 7 studies showed significantly higher mortality in NAAT+/EIA+ patients, with no difference in all-cause mortality found in that study (16). Fulminant CDI was also seen at comparable proportions in NAAT+/EIA− and NAAT+/EIA+ patients, underscoring that NAAT+/EIA− patients cannot be assumed to be colonized solely based on their negative toxin assay results. Colectomies were no more frequent among NAAT+/EIA+ patients in institutions implementing a two-step testing algorithm, suggesting that this rare complication cannot be used as an indicator that CDI treatment may be safely withheld from toxin-negative patients (33, 77).

Overall, more than 70% of NAAT+/EIA− patients received treatment for CDI. This high proportion, along with the fact that nearly all tested patients regardless of EIA status complained of diarrhea, demonstrates that it is not accurate to state that a negative EIA result necessarily indicates that colonization is likely, as some have claimed (78). Likewise, it is not accurate to assume that toxin positivity is necessarily indicative of CDI, as asymptomatically colonized individuals may also be EIA+ (51). The proportion of EIA− patients who are treated for CDI varies at different institutions and will be influenced by the stringency of criteria for testing and the manner in which results are reported (77). Reporting practices vary among institutions, with some interpreting the result as “consistent with colonization or infection” while others interpret a negative EIA to mean “likely colonization.” One recently published study from Turner et al., in which NAAT+/EIA− results were reported as “likely colonization,” was an outlier in our analysis of the pooled likelihood of antibiotic treatment in NAAT+/EIA− patients (33). Only 16% of patients in that study were treated for CDI (33), in contrast to the pooled likelihood of 72.4% from 19 studies (Fig. 5). Patient-level data regarding clinical outcomes of untreated NAAT+/EIA− patients were not provided in the study by Turner et al. Accordingly, it is important to mention that it has not been shown that antimicrobial therapy may be safely withheld from symptomatic EIA− patients, and current recommendations are to base treatment decisions on clinical assessment rather than toxin positivity (79). In contrast to Turner et al. (33), a recently published CDC-sponsored multicenter study found that approximately 95% of patients with NAAT+ stools had diarrhea, and more than 70% of the patients with NAAT+/EIA− results received CDI treatment after clinical assessment, indicating that a NAAT+/EIA− result in a symptomatic patient most likely represents CDI (28). These findings are consistent with the results of the present meta-analysis. However, this does not exclude the possibility that antibiotics might have been unnecessary in some NAAT+/EIA− patients who received CDI treatment. A small number of studies that assessed outcomes of NAAT+/EIA− patients did not find an increase in adverse outcomes in untreated patients at 30 days or 8 weeks (16, 58, 80). On the other hand, severe CDI complications have been reported in NAAT+/EIA− patients (28, 44, 49, 55, 56, 71), and some NAAT+/EIA− patients have progressed to require initiation of treatment and ICU admission due to CDI (44, 58), demonstrating that a NAAT+/EIA− result in a symptomatic patient does not always predict a benign clinical course. In addition, some patients with CDI may experience the resolution of symptoms after discontinuation of the inciting antibiotic without specific CDI treatment.

This meta-analysis was not able to show a difference in mortality between treated and untreated NAAT+/EIA− patients, in contrast to another recent meta-analysis by Prosty et al. that found all-cause mortality to be significantly lower in treated NAAT+/EIA− patients (81). This discrepancy is attributable to the inclusion of unpublished data in the study by Prosty et al. (81), whereas only published data were included in our meta-analysis. Furthermore, patients who receive treatment may be sicker, and treatment may contribute to a reduction in their mortality, such that their outcomes become comparable to those of untreated patients with less severe CDI.

When NAAT+ or toxigenic culture-positive/EIA− patients were compared to EIA+ patients in terms of mortality and recurrence, outcomes were not significantly different between the two groups. This may be attributed to the relatively small number of patients included in these analyses, although the results are comparable with what was observed in NAAT+/EIA− vs NAAT+/EIA+ patients. As NAAT and toxigenic culture have similar sensitivity (82), these findings are not unexpected. Moreover, NAAT+ and EIA+ patients exhibited a similar risk of mortality or recurrence, but the sample size was small.

Conventional EIAs are insensitive for the detection of *C. difficile* toxins (11, 83). New ultrasensitive toxin assays have been developed with much higher sensitivity for *C. difficile* toxin detection (51, 84). When a more sensitive assay is employed, most EIA− patients are actually toxin-positive, indicating that EIA− patients have lower rather than absent levels of toxin in their stool (51). Nevertheless, even ultrasensitive toxin assays are unable to distinguish patients with asymptomatic *C. difficile* carriage from those with CDI, since low toxin levels are sufficient to cause disease, and high toxin levels can be seen in some asymptomatically colonized individuals (51, 85, 86). Interestingly, inflammatory biomarkers appear to better distinguish colonization from disease, and future tests combining pathogen detection with indicators of inflammation may improve diagnostic accuracy for CDI (87, 88). As many as half or more of patients with symptomatic CDI are EIA-negative (56, 63, 66, 71). Importantly, toxin-negative patients with CDI respond similarly to treatment (89, 90), and fulminant colitis leading to colectomy may still occur in EIA− patients (91).

Despite the increasing use of more sensitive NAAT assays, rates of hospital-onset CDI in the USA declined during 2011–2017 following the widespread implementation of control measures (2). A recent genomic surveillance study found in-hospital *C. difficile* acquisition to be uncommon, with most cases of CDI observed in patients with pre-existing toxigenic *C. difficile* colonization (92). These data question whether crude CDI rates have outlived their usefulness as an indicator of the efficacy of infection prevention measures. Future preventive measures may involve screening, more judicious use of broad-spectrum antibiotics, or possibly prophylaxis (e.g., probiotics [93], fidaxomicin [94]), as CDI in colonized individuals will not be preventable by isolation and contact precautions.

A major driver of CDI testing has been NHSN reporting requirements. However, the clinical diagnosis of CDI cannot be solely based on laboratory tests (9). For institutions performing a two-step testing algorithm, it is critical that their clinicians understand that discordant NAAT+/EIA− results can represent either colonization or infection, with clinical correlation required to make the distinction. The NHSN is aware that two-step algorithms underdiagnose CDI and is in the process of revising the definition of healthcare facility-onset CDI from exclusively lab-based criteria to a combination of lab results and antibiotic treatment (“healthcare facility-onset, treated CDI”) (77, 95). As the majority of NAAT+/EIA− patients receive CDI treatment, it can be predicted that a change in the NHSN definition will result in higher reported CDI rates in US healthcare institutions. Microbiology laboratories must also decide whether to report *C. difficile* results from multiplex PCR panels. One study has shown that blinding these results can result in missed CDI diagnoses and serious complications (96).

A major strength of this study is the sample size. This is the largest meta-analysis of CDI patient outcomes based on toxin EIA result and the first to assess the risk of complications. Another strength is the pooled assessment of treatment likelihood in NAAT+/EIA− patients, which was found to vary substantially among institutions and is an important factor for institutions performing two-step testing algorithms to consider. A third strength is that despite differences in the definition of CDI complications in various studies, only studies with consistent definitions were pooled. Important limitations of the study must also be acknowledged. None of the included studies was randomized, resulting in a potential for confounding bias, which was not assessed. In addition, most of the studies had a retrospective study design; therefore, the quality of the included data (e.g., assessment of clinical outcomes) may not be optimal. Lastly, the results of this analysis may have been influenced by study heterogeneity including differences in the time periods, patient population, diagnostic indications, and treatment strategies that might have influenced clinical outcomes. For example, metronidazole was formerly used as a first-line agent for mild-to-moderate CDI but is no longer recommended for this purpose (15).

In conclusion, this systematic review and meta-analysis concludes that NAAT+/EIA− patients have similar risk of mortality and CDI-related complications to that of NAAT+/EIA+ patients but a lower risk of recurrence and severe CDI. The toxin result should not be a determining factor in the administration of CDI therapy, as a negative toxin assay cannot rule out CDI, and a positive toxin assay cannot rule it in. Most symptomatic patients with a negative toxin result are still likely to have CDI rather than colonization and may benefit from treatment (79). Consequently, laboratory reporting of discordant results by a two-step algorithm should be agnostic and emphasize that treatment decisions are to be based on clinical assessment. Toxin testing can help to predict the likelihood of CDI recurrence or severe infection. Future studies may evaluate the clinical significance of different preventive and therapeutic approaches (e.g., use of alternative regimens of fecal transplantation) in patients with higher risk of recurrence.

## SUPPLEMENTAL MATERIAL

Figure S1 jcm.00977-24-s0001.tif Fulminant CDI.

Figure S2 jcm.00977-24-s0002.tif Total complications.

Figure S3 jcm.00977-24-s0003.tif Colectomies following CDI.

Figure S4 jcm.00977-24-s0004.tif Radiographic evidence of CDI.

Figure S5 jcm.00977-24-s0005.tif ICU admission.

Figure S6 jcm.00977-24-s0006.tif Attributable mortality.

Figure S7 jcm.00977-24-s0007.tif Receipt of antibiotics.

Figure S8 jcm.00977-24-s0008.tif All-cause mortality in NAAT+ or toxigenic culture+/EIA- vs EIA+.

Figure S9 jcm.00977-24-s0009.tif Recurrence in NAAT+ or toxigenic culture+/EIA- vs EIA+.

PRISMA checklist jcm.00977-24-s0010.docx PRISMA checklist.

ASM does not own the copyrights to Supplemental Material that may be linked to, or accessed through, an article. The authors have granted ASM a non-exclusive, world-wide license to publish the Supplemental Material files. Please contact the corresponding author directly for reuse.
