# Supplementary figures and images for: Clinical significance of toxin EIA positivity in patients with suspected Clostridioides difficile infection: systematic review and meta-analysis
Source: J Clin Microbiol. 2024 Dec 12;63(1):e00977-24. doi: 10.1128/jcm.00977-24 (PMC11784090; doi:10.1128/jcm.00977-24)

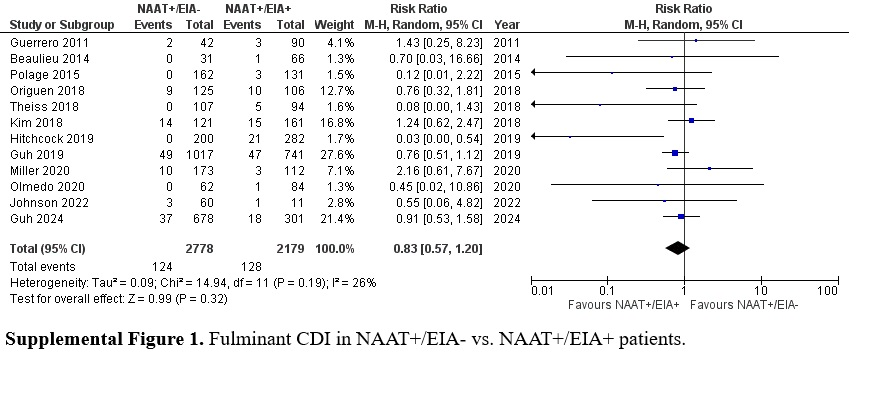

Supplement: Figure S1 — Fulminant CDI. [file jcm.00977-24-s0001.tif]

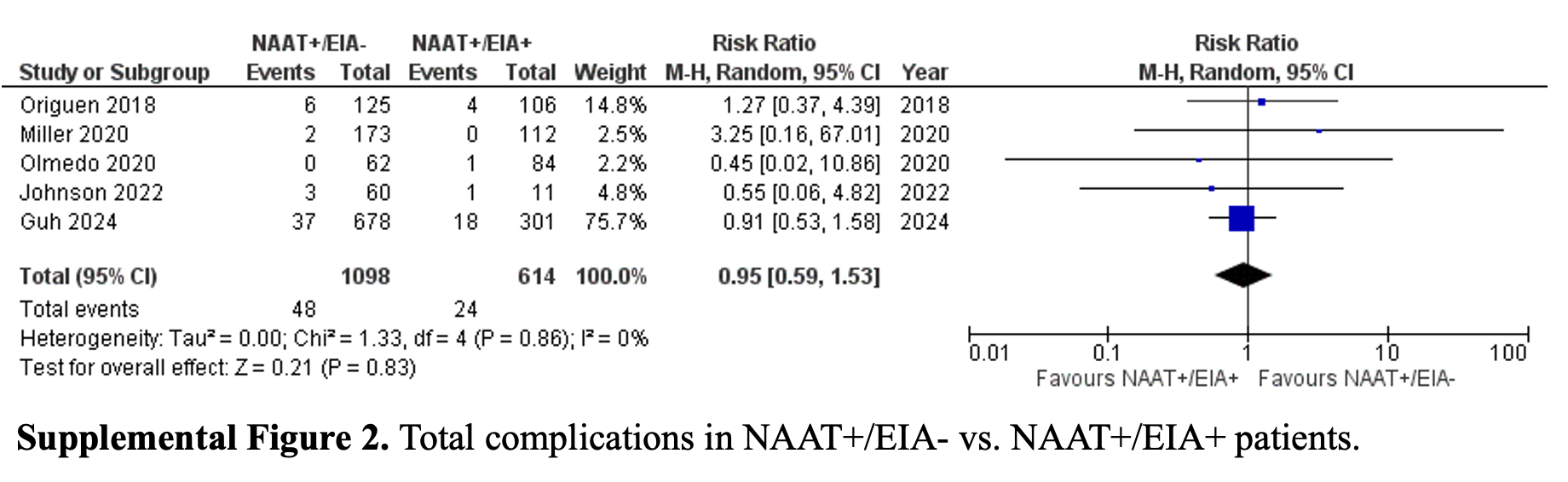

Supplement: Figure S2 — Total complications. [file jcm.00977-24-s0002.tif]

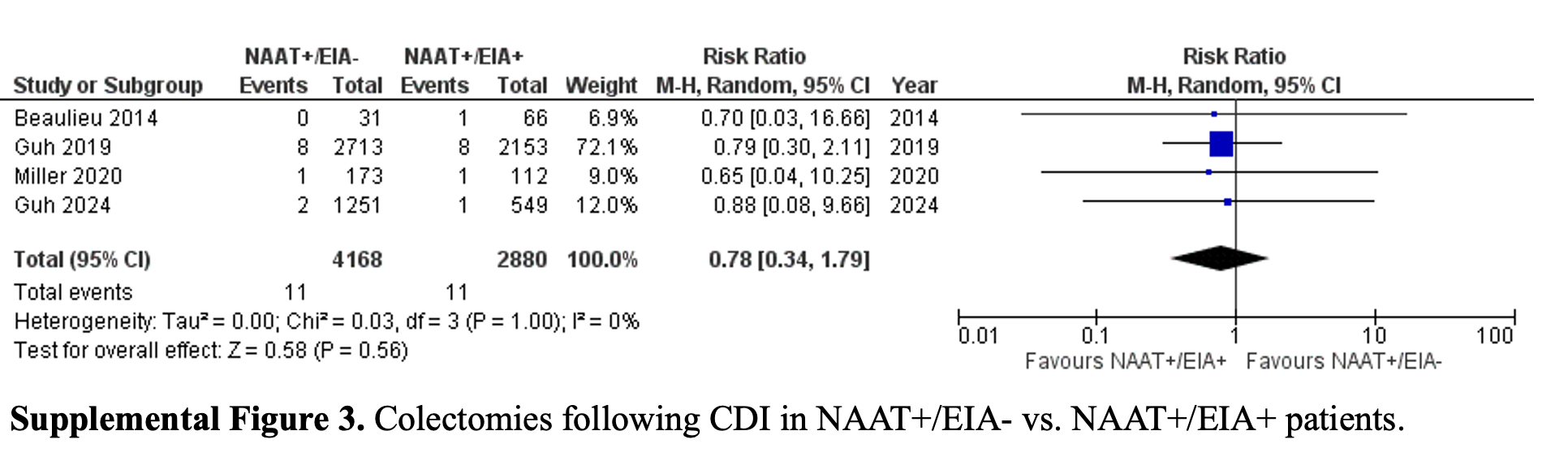

Supplement: Figure S3 — Colectomies following CDI. [file jcm.00977-24-s0003.tif]

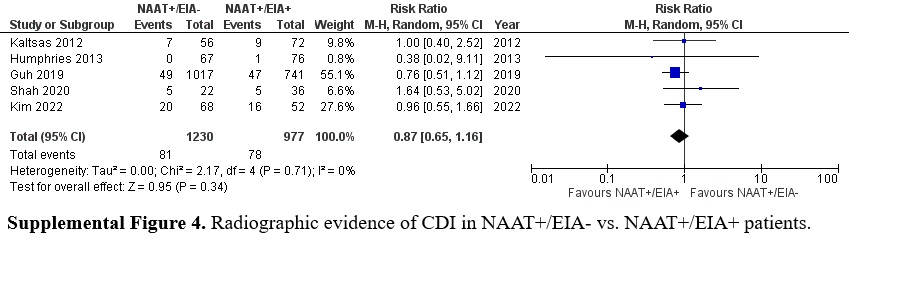

Supplement: Figure S4 — Radiographic evidence of CDI. [file jcm.00977-24-s0004.tif]

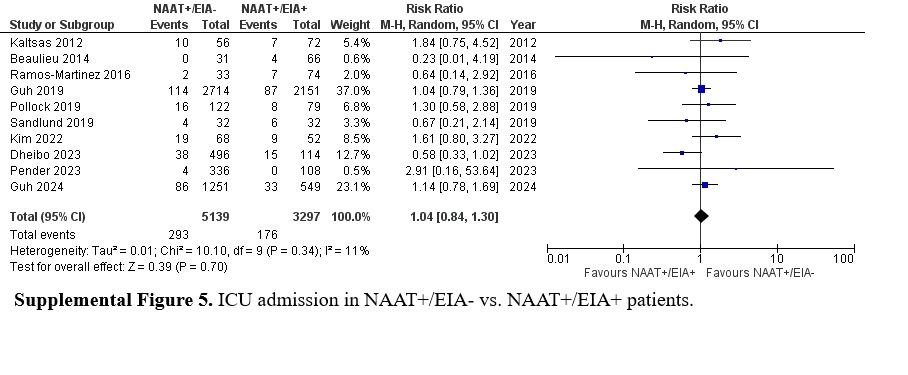

Supplement: Figure S5 — ICU admission. [file jcm.00977-24-s0005.tif]

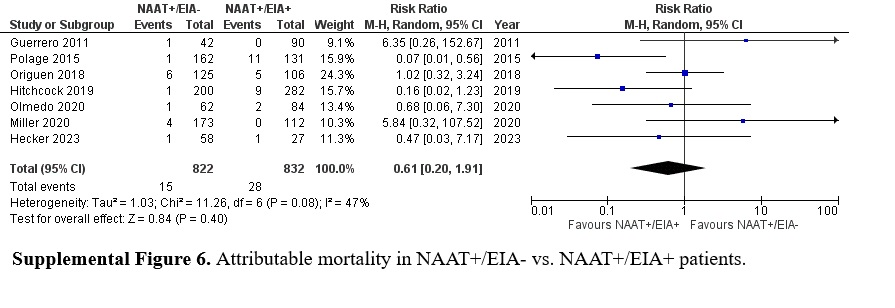

Supplement: Figure S6 — Attributable mortality. [file jcm.00977-24-s0006.tif]

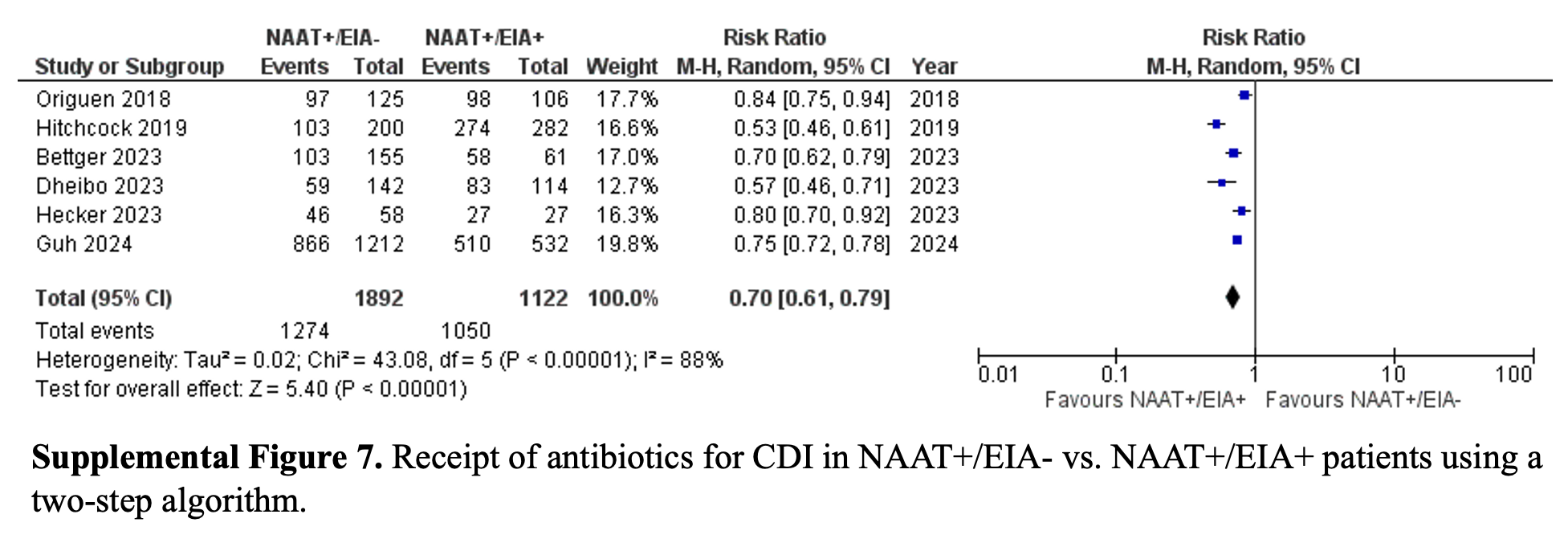

Supplement: Figure S7 — Receipt of antibiotics. [file jcm.00977-24-s0007.tif]

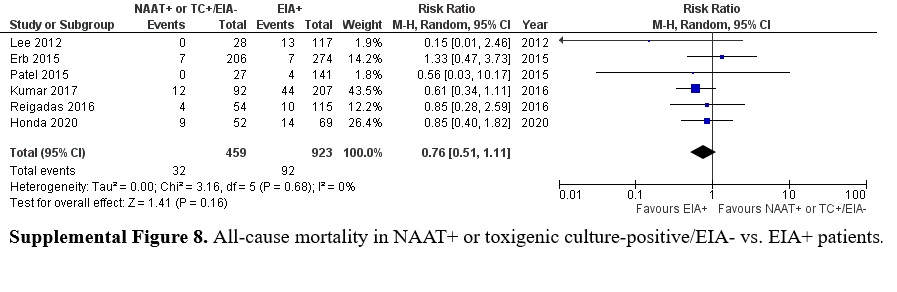

Supplement: Figure S8 — All-cause mortality in NAAT+ or toxigenic culture+/EIA- vs EIA+. [file jcm.00977-24-s0008.tif]

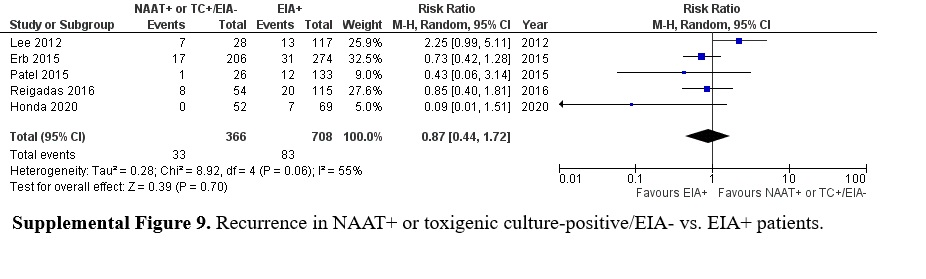

Supplement: Figure S9 — Recurrence in NAAT+ or toxigenic culture+/EIA- vs EIA+. [file jcm.00977-24-s0009.tif]
